# “I Am Forever Changed”: A Phenomenological Study of the Existential Experiences of Parents of Children and Young People With Complex Care Needs

**DOI:** 10.1111/jan.16509

**Published:** 2024-10-02

**Authors:** Carina Nygård, Anne Clancy, Gabriele Kitzmüller

**Affiliations:** ^1^ Department of Health and Care Sciences, Faculty of Health Sciences, UiT The Arctic University of Norway Harstad Norway; ^2^ Department of Health and Care Sciences, Faculty of Health Sciences UiT, the Arctic University of Norway Narvik Norway

**Keywords:** children, complex care needs, existential, family health, lived experience, long term illness, nursing, parents' experiences, phenomenology

## Abstract

**Aim:**

To explore the existential lived experiences of parents of children and young people with complex care needs.

**Design:**

An explorative qualitative design.

**Methods:**

We conducted 16 in‐depth interviews with parents of children and young people with complex care needs across Norway from February to May 2022. Data collection and analysis were guided by the theoretical framework of van Manen's phenomenology of practice approach.

**Results:**

Four distinct but interwoven themes reflecting the comprehensive and holistic nature of parents' existential lived experiences emerged: lived body: “I am forever changed”; lived space: Seeking sanctuary; lived time: “Time doesn't seem to exist”; and lived self‐other: Parents' changing relationships.

**Conclusions:**

Parents' ongoing efforts to manage fluctuations in their daily lives profoundly affect the existential aspects of their well‐being, suggesting that respectful and attentive nurse–parent relationships can nurture existential growth.

**Implications for the Profession and Patient Care:**

There is a crucial need for a genuine nursing presence characterised by an attitude of open sensitivity and attentive listening to parents' existential experiences. Nurses should embrace the opportunity to engage in respectful and attentive dialogues with parents. Acknowledging that the insights emerging from these conversations can improve integrated and personalised nursing services worldwide.

**Impact:**

Parents of children and young people with complex care needs often experience suboptimal healthcare. Additionally, access to quality healthcare services, particularly in rural areas, is limited, creating inefficiencies and coordination challenges. This study provides nurses, other health care professionals, researchers, and decision‐makers with valuable perspectives on supporting parents' existential needs which may significantly impact their overall well‐being and coping abilities, contributing to a more compassionate approach to family care.

**Reporting Method:**

Consolidated Criteria for Reporting Qualitative Research (COREQ).

**Patient or Public Contribution:**

Parents of children and young people with complex care needs provided valuable feedback on the findings and implications of this work.

**Summary**

- What does this article contribute to the wider global clinical community?
  ○ Parents of children and young people with complex care needs shoulder enormous responsibilities and express the need for well‐coordinated services, appropriate information, and emotional and practical support.
  ○ Nurses must recognise the wide range of existential needs of parents of children and young people with complex care needs and understand how they manifest to provide high‐quality care.
  ○ As paediatric care shifts from the hospital to home settings, clear guidelines and framework conditions that enable nurses to address the existential needs of parents as care providers become necessary.

## 1 Introduction

Globally, children and young people with complex care needs (CCNs) constitute a diverse and growing population requiring extensive health and social care due to a comorbidity of conditions (Azar et al. 2020). Their caring needs are highly personalised, continuous, and dynamic, often shaped by their unique family situations and the healthcare system (Brenner et al. 2018). These needs are often best served by integrated and tailored health care services available close to the family's home (Cassidy et al. 2023). The prevalence of children and young people with CCNs varies, influenced by differing definitions; however, a recent US estimate suggests that approximately 25% of children up to 18 have CCNs (Yang et al. 2024).

Parents as primary caregivers play a significant role in managing the care of their children or young people and often become experts at monitoring their basal needs and clinical deteriorations (Nygård and Clancy 2018). While parents' self‐efficacy and self‐management have been linked to an enhanced quality of life (Genna et al. 2023), they continue to endure a high level of parenting pressure, stress, and inadequate support, which profoundly impacts their physical and emotional wellbeing (Yang et al. 2024). Simultaneously, exhaustion can diminish their caregiving capacity and potentially worsen the child's, young person's, and the overall family's wellbeing (Cohn et al. 2020). Therefore, it is crucial to understand the unique needs of these parents, including how to alleviate their burden, to ensure that care and support are effective and meet their individual and family needs.

The family‐centered care approach focuses on collaboration between families, children, and nurses to enhance family well‐being and requires understanding parents' perspectives on their caregiving roles (Almasri, An, and Palisano 2018). However, despite extensive research, the concept of family‐centered care remains ambiguous, resulting in uncertainties in its implementation (Kokorelias et al. 2019).

## 2 Background

Previous reviews have documented the profound impact of a child's CCNs on parents (Cohn et al. 2020; Leite et al. 2019; Nygård and Clancy 2018). This includes managing intricate care procedures and treatments and coordinating necessary health and social services, all while adapting to an unusual family situation (Currie and Szabo 2019).

Daily uncertainties can impact parents' well being as they strive to maintain a “day‐to‐day” balance, reaffirming hope for the future (Leite et al. 2019). Parents often experience emotional and physical strain, including anxiety, depression (Cohn et al. 2020), frustration, anger (Leite et al. 2019), worrying, pain, sleep disturbances, and fatigue (Dlamini, Chang, and Nguyen 2023). These health concerns may be accompanied by existential feelings of loss, guilt, loneliness, and hopelessness (Leite et al. 2019). While these existential experiences are universal (van Manen 2023), parenting children with CCNs can lead to existential suffering (Dlamini, Chang, and Nguyen 2023). However, some parents also report that children's illness can strengthen family bonds and foster personal growth (Nygård and Clancy 2018).

Undeniably, parenting children with CCNs is a major responsibility that often limits parents' access to respite care (Murphy et al. 2022). Although they may benefit from respite care, parents often hesitate to use it due to concerns about the competence of healthcare providers, as healthcare professionals are not always responsive to parents' needs, underscoring the need for nurses trained in paediatric specialist care (Murphy et al. 2022). Identifying how to implement competent care for families raising children with CCNs calls for updated research that offers insights into what it *means* to be a parent to such children.

The use of phenomenological approaches in qualitative nursing research offers comprehensive insights into caregivers' lived experiences, revealing how they can contribute to optimising existential care for this vulnerable population. However, the existential experiences of parents caring for children with CCNs remain underexplored.

Existential care, recognised as a fundamental for nursing, is centered on how individuals derive meaning from their life situations (Nygaard et al. 2022). The prevalent use of phenomenological approaches in qualitative nursing research sheds light on improving existential care for vulnerable populations. However, the expression of these needs in the daily life of parents of children with CCNs often goes unnoticed by nurses in clinical settings (Nygård and Clancy 2018).

## 3 The Study

The current study explores the existential lived experiences of parents caring for children and young people with CCNs. Specifically, it addresses the following phenomenological research question:

What are the existential experiences of parents who care for children and young people with CCNs?

## 4 Methods

### 4.1 Design

This study employed a hermeneutical phenomenological methodology within van Manen's theoretical framework of “phenomenology in practice” (2023), widely used in nursing science to explore sensitive aspects of human life experiences (Errasti‐Ibarrondo et al. 2019). Following van Manen (2017, 812), “lived experiences” refer to everyday “raw” occurrences that engender specific situations. Yet, the moment we attempt to describe these experiences, they have already passed (van Manen 2017). Hence, adopting a phenomenological attitude is essential for this inquiry (van Manen 2023). An in‐depth exploration and sensitive interpretation of these moments aim to uncover the initial meanings tied to the experience of parenting children with CCNs (van Manen 2017, 819). Language plays a pivotal role in this research design as engaged and thoughtful writing aids in the reflective and interpretive process, providing insights that enable readers to revive and comprehend the experience (van Manen 2023).

### 4.2 Study Setting and Recruitment

Although Norway's publicly funded healthcare system offers universal coverage, access to services vary widely across settings and regions, resulting in significant disparities on both national and international levels (Cassidy et al. 2023; Office of the Auditor General of Norway 2021). Despite this and in line with international trends, parents shoulder the primary caregiving burden, yet their involvement in health policy development is minimal (Brenner et al. 2018).

We utilised snowball sampling, effective in accessing hard‐to‐reach populations (Noy 2008), to recruit a diverse group of Norwegian parents of children and young people with CCNs, to capture varied caregiving experiences and geographic diversity. Initially, we used our own networks to find people acquainted with potential participants, who then referred other parents. The study's inclusion criterion was being a mother or father of a child or young person with CCNs, meeting the characteristics of children and young people with CCNs specified in Brenner et al. (2018, 1647). In this study, children and young people were defined as those under 18 (United Nations 1989) and between 15 and 24 years old (United Nations 1985), respectively. The paediatric age range may vary by the healthcare system and country; however, paediatric care generally caters to the caring needs of children and young people up to the age of 18 or up to 21 for those with CCNs (Hardin et al. 2017). Parents were eligible for inclusion, regardless of their geographic residence, marital status, or employment status. We selected parents of children and young people up to age 20 living at home to capture a wide range of parenting experiences and perspectives. This age group encompasses various stages of childhood and adolescence, providing a comprehensive understanding of the essence of parenting children and young people with CCNs. We excluded parents caring for their children and young people at the end of life, acknowledging the distinct existential experiences associated with terminal care. Interested participants contacted the first author via email or phone for further information. This referral process continued until we achieved diverse data material. None of the participants declined to participate in the study. The authors did not offer any incentives for participation.

### 4.3 Gathering Lived Experience Descriptions

Between February and May 2022, the first author, a nursing researcher with extensive clinical experience, conducted face‐to‐face interviews with parents to obtain detailed descriptions of their lived experiences, through eliciting anecdotes and meaningful stories (van Manen 2023). Based on van Manen's (2023) life world existentials (corporality, temporality, spatiality, and relationality) and drawing on our own expertise and knowledge in the field, we sought to facilitate a natural conversation that allowed parents to express themselves freely. We predetermined a set of questions to prompt reflection and ensure that the interviews captured concrete stories of significant moments of parents' life, allowing the phenomena to unfold. To gain a deeper understanding of the meanings embedded within their stories, follow‐up questions were asked.

Each interview began with questions that were rooted in the parents' unique contexts, from which their experiences derived meaning (Bevan 2014). Hence, we asked for descriptions of being a parent to a child and young person with CCNs and about significant moments or settings parents found challenging or fulfilling. We were particularly interested in understanding how these parents experienced typical family situations such as weekends, leisure times, holidays, and mealtimes. As the interview proceeded, we allowed the parents the opportunity to share more intimate aspects of their experiences. Recognising that an experience can be viewed from multiple perspectives, we asked diverse questions to comprehend the nature of a specific experience (Bevan 2014). Table 1 summarises the interview questions. Immediately after each interview, the first author documented field notes capturing subjective impressions about the emotional tone and non‐verbal behaviour observed during the interview, as well as personal reflections on the interview process.

**TABLE 1:** Summary of interview questions.

Questions framing the parents' unique contexts
Can you tell me about yourself? Name, family, home, occupation? Could you briefly describe your child's complex care needs? How would you describe your daily life?
Questions to comprehend the nature of a specific experience
Can you tell me about a situation from the past week that brought you joy? Can you recall a time when you were especially saddened because your child or young person needed more help than their healthy counterparts? How did you handle those feelings? Could you describe your relationship with your spouse and your other children? Could you describe times when you can focus solely on yourself? If you needed support, who would you turn to? What are your thoughts on the quality of the healthcare services and specifically the nursing services that you receive? How can the healthcare system be improved? How do you see the future?

The interviews were conducted either in their homes or at the university, according to the parents' preference. Due to the COVID‐19 pandemic, six interviews were conducted using the Teams video‐conference platform. This remote approach to data collection broadened the geographical scope of participant inclusion.

The notion of data saturation does not align with hermeneutic phenomenological methodology (van Manen 2023). During the interviews, parents provided a broad range of significant experiences from their daily lives. Based on their duration and depth, 16 interviews were deemed sufficient to yield a rich dataset that would provide meaningful insights relevant to the study's objectives (van Manen 2023). The interview recordings lasted between 1.5 and 3 h; they were anonymized and transcribed verbatim by the first author to ensure data accuracy.

### 4.4 Data Analysis

van Manen's thematic phenomenology of practice approach (van Manen 2023) was applied to the transcriptions to generate deeper insights into and understanding of the lived meaning of parenting children and young people with complex needs. van Manen (2023) contends that articulating meaningful structures is never a straightforward process but requires thoughtful reflection and interpretation that can reflect the immense complexity of different lifeworlds. When attempting to understand the meaning of a phenomenon, one can easily get lost or become anchored in certain aspects, due to its complexity (van Manen 2023). To ensure clarity, themes were classified according to van Manen's (2023) four existential dimensions of lived space (spatiality), lived body (corporality), lived time (temporality), and lived self‐others (relationality). Although these dimensions are interwoven and mutually influential, they constitute the fundamental aspects of human existence. Lived body relates to how we are bodily engaged in the world, lived space refers to how we attach meanings to different spaces we occupy, lived time captures our reflections on how we experience our time, and lived self‐other describes how we experience ourselves in relation to others (van Manen 2023).

Early in the analysis, we employed a holistic reading approach by reading each interview several times to gain a sense of the whole picture. The first author examined one lifeworld existential dimension at a time as a lens on each of the 16 interviews. No preference was given to transcripts or existential dimensions; all were approached equally. Data immersion, however, revealed that the existential dimension of corporality and relationality dominated the descriptions. Then, a selective highlighting reading path was followed, during which sentences, statements, and keywords related to meaningful aspects of parents' lived experiences were marked and formulated as initial themes (van Manen 2023). Within each existential dimension, similar themes from the interviews were identified. Instead of adhering to a predetermined approach, essential themes were developed through an iterative process characterised by common reflective discussions, writing, and rewriting the text to uncover the core of the parents' lived experiences. Writing served as a tool for gaining perspectives, which facilitated the cultivation of thematic insights (van Manen 2023). To reach consensus on the final themes, the authors engaged in extensive discussions about the variations within each essential theme and its relevance to the research question. Ultimately, we agreed on four final themes that best represent the existential experience of parenting children and young people with CCNs.

### 4.5 Ethical Considerations

The Norwegian Agency for Shared Services in Education and Research approved the study under registration number 601507. According to the Norwegian Regional Committee for Medical Research Ethics, the study is not covered by the Health Research Act (journal number 32053); thus, further approval was not required. Participants received information about the study and provided their oral and written informed consent, with the assurance that they could withdraw at any time prior to publication. We promised the participants confidentiality. Hence, the data collected were password protected, encrypted, and stored securely. Prior to data collection, a distress protocol was developed to identify and guide immediate stress responses. However, the interviewer, a nurse trained to handle emotional reactions, was attentive to signs of severe distress in participants, but none were observed.

### 4.6 Rigour and Reflexivity

We adhered to Lincoln and Guba's (1985) criteria of credibility, transferability, dependability, and confirmability to ensure research rigour. The authors are all female nurses with extensive experience caring for patients and their families. Each holds academic positions in nursing research and education. The first author is a doctoral student while the second and third authors are senior researchers. Despite the authors not being involved in direct care and the first author, who conducted the interviews, not having a prior relationship with the parents, preconceptions are an inherent part of understanding. Nevertheless, throughout the research process, we maintained an open attitude of curiosity, not taking any insight for granted (van Manen 2023). By questioning assumptions and engaging in collaborative discussions, the authors identified blind spots that fostered critical thinking, broadened our perspectives, and deepened our understanding of the research questions.

To demonstrate dependability and transferability, we strove to maintain an audit trail with detailed descriptions at every methodological step. Original statements from parents were used to illustrate the themes, and situated descriptions illuminated the richness and variety of human experience (van Manen 2023), establishing confirmability. The involvement of a user group representing parents of children and young people with CCNs provided valuable feedback on the study's findings and clinical implications to strengthen the study's credibility. We adhered to the Consolidated Criteria for Reporting Qualitative Research (COREQ) checklist (Appendix S1) (Tong, Sainsbury, and Craig 2007).

## 5 Findings

Two fathers and fourteen mothers—all biological parents and primary caregivers of children and young people with CCNs between 6 months and 20 years—were eligible for study inclusion (Table 2). Only two of these were single parents. Except for one, all participants were raising multiple children.

**TABLE 2:** Participant characteristics.

Participant/ID	Gender	Child's diagnosis	Child's age
1	Female	Genetic disorder	1,5 years
2	Female	Congenital heart defect, heart transplant	13 years
3	Female	Cleft lip and palate, epilepsy	13 years
4	Female	Leukaemia, lymphoma cancer	13 years
5	Female	Diabetes 1	14 years
6	Male	Diabetes 1	11 years
7	Female	Premature, cerebral palsy, genetic disorder	6 months
8	Female	Juvenile rheumatoid arthritis	12 years
9	Female	Downs syndrome, congenital heart defect	6 years
10	Female	Autism and diabetes	13 years
11	Female	Premature, multiple developmental delays	17 years
12	Female	Diabetes	17 years
13	Female	Epilepsy, genetic disorder	13 years
14	Male	Autism	5 years
15	Female	Diabetes 1, celiac disease	13 years
16	Female	Autism	20 years

The analysis uncovered four phenomenological themes that capture aspects of the parents' lived experiences when caring for children and young people with CCNs. These themes explicate how the four existential dimensions of lived body, lived time, lived space, and lived self‐other may provide core insights that constitute parents' lifeworlds. The reader may recognise the essence of their lived experiences, described by van Manen (2023) as the phenomenological nod.

### 5.1 Lived Body: “I Am Forever Changed”

The theme of corporality refers to the parents' experiences of bodily existence, including the sensation of having their awareness of being in a physical body. In the parents' narratives of their daily lives, these embodied perceptions are complementary and interrelated, reflecting the varied and dynamic nature of their existential lifeworld.

Subtle existential feelings such as inadequacy, helplessness, emptiness, and unfamiliarity were obvious in all interviews. Parents spoke of being overwhelmed with guilt, anxiety, frustration, fear, sorrow, a profound sense of loneliness, and isolation. These emotions contributed to feeling estranged from their former selves as parents: “The life you once knew is over. Now there seems to be nothing left” (ID 4).

They frequently conveyed a sense of entrapment, caught in a situation that greatly limited their freedom and control. They sought out information and tried to modify their parenting strategies. Yet, these efforts often proved fruitless, exacerbating feelings of helplessness, sadness, loss of confidence, and longing for time beyond the confines of caregiving:

I am almost just a mom. It does not suit me. It is not that I do not love my kids; I am good at being a mom. But I do not like it. I do not like being just that. There must be something more. (ID 1)

Falling short of their own hopes could lead to chronic sorrow, threatening their existence. Sadness and grief often became overwhelming, as they mourned their losses. Some parents yearned to know the people their children would have become had it not been for their illnesses, reflecting on how the condition had changed their children's potential for normal development:

The most challenging thing was losing my little boy. So, my grief doubled. First, I had to come to terms with him getting sick and losing himself. Then, there is sorrow over losing everything that could have been. I could never be without the time we had together when he was healthy. That is the hardest part. (ID 14)

Another profound sorrow shared by the parents was the loss of their once healthy bodies. They spoke of how physical and emotional strains affected their physical health. Perceived muscle tensions, accelerated heart rates, and tiredness were inextricably linked to the intense situations these parents navigate, signalling stress, fatigue, and the need for respite. Parents of children with severe complexities, such as multiple medical conditions or genetic and neurological diseases, described a deep alienation from their own physicality, experiencing a dissonance between their changing bodies and their sense of self. A father elaborated: “Before, my body and temperament were all in harmony. I am forever changed” (ID 14).

Most parents conveyed a sense of bodily weakness, feeling physically exhausted by even the smallest tasks. Their reduced energy implied a decline in personal freedom, especially regarding engaging in life beyond the confines of their home:

There is not as much energy to do other things as there used to be. For instance, if I go skiing on a day when the sun is shining and the snow is sparkling, I may feel tired because I have slept poorly the last few days. Then I end up lying on the sofa instead, whereas before I would have run outside. (ID 6)

Over time, the presence of a fatigued body became increasingly apparent, accompanied by an overwhelming feeling of powerlessness that prompted existential reflections such as: “My body already feels aged, and I wonder how long I would be able to withstand this situation” (ID 1). The situation culminated for a mother with an ill baby who was unable to fall asleep: “I was about to lose it. I yelled and my husband took over” (ID 7).

Although parents tried to conserve their strength, fatigue persisted, often intensifying at night when intrusive thoughts kept them awake. Even on nights when their children did not require immediate care, many parents lay awake, on high alert for any alarms or acute illness changes. This pattern of disrupted sleep, persisting over time, could have significant repercussions on their health, as one mother described:

I simply became burnt out from it all. For many years, I did not sleep. There is just no time for sleep. I have been on night shift after night shift; we have fought and struggled with everything. In the end, it just completely hit me, and I was totally flattened. (ID 13)

Despite their diminished strength and vitality, the parents had no choice but to continue caregiving. A mother said: “If I fall apart, she falls apart too. It is not just about her; it is about the whole picture. We need to function. So, if I hit a wall, it will affect everyone” (ID 5).

Nonetheless, parents felt the full weight of responsibility on their shoulders to meticulously manage their children's needs. Anxiety caused considerable emotional distress, often mixed with worry and self‐blame. Although parents expressed confidence in their ability to notice significant changes in their children's condition, they were anxious about overlooking or underestimating symptoms: “Like, when we go to the eye doctor and he says, ‘You should have been here three months ago’.” (ID 1). This constant burden not only made respite difficult but also resulted in tangible physical stress reactions:

I was out skiing, and suddenly, it just hit me. I could not … I thought, “I am going to die.” My first thought was that I was having a heart attack, but then I switched to a more rational part of my brain, lay down, and tried to catch my breath. That is when I started struggling with a sort of anxious feeling in my body. (ID 4)

A profound sense of guilt emerged when parents spent time away from their ill children during vacation. The fear of what might occur in their absence prevented them from being fully present in their surroundings, as they continually focused on the well‐being of their ill children:

We were supposed to go on holiday, so I could take care of my other children. I think I started crying at eleven in the morning. I just kept on crying, Non‐stop crying. I could not relax. (ID 4)

With time, most parents adjusted to the situation, developing a sense of autonomy and mastery, which made them more resilient. However, they remained constantly vigilant, with their mobile phones serving as an extension to their bodies, always prepared to respond to ensure their children's well‐being.

Despite the limitations, parents' existential experiences enhanced their self‐awareness, contributing to a deeper understanding of the opportunities their current life presented. A sense of inner contentment could also emerge from the acceptance that they, despite all the hurdles, had optimised their situation.

### 5.2 Lived Space: Seeking Sanctuary

Parents' experiences of their lived space in the various settings of everyday life enhanced an understanding of how they perceived different spaces as either limiting or freeing. These experiences evoked a range of emotions that profoundly impacted parents' well‐being. Parents' homes served as an anchor of stability, continuity, and control.

At home, we have much more control, and we have our own routines. We lock the kitchen and clear away things that can be knocked over—liquid soap dispensers, flower vases, and candle holders. (ID 16)

Homes were not just physical locations but sanctuaries providing them with a sense of freedom and autonomy, contributing to a feeling and place of belonging. In the familiar and intimate setting of their homes, parents felt free from the constraints of the outside world and most confident in their caregiving role:

We were completely exhausted after the hospital stay. We were very eager to get home and out of that system. (ID 7)

While most parents considered their homes havens of strength, those with severely ill children described their homes as confining and restrictive, inducing feelings of isolation. Thus, the necessity of meeting their children's needs had a detrimental impact on parents' personal space and overall well‐being. The presence of medical equipment and a continual flow of homecare providers threatened parents' dignity, leaving them feeling exposed and struggling to maintain control over their own home. Although all parents valued the support received from homecare providers, the need to give them access to their home, so that they could come and go as needed, was an invasion of privacy for some:

Of course, you are grateful for the help. It is just that at night you cannot just run to the bathroom in your underwear or anything like that because there could be someone there. You do feel a bit invaded, but you do not have a choice. (ID 11)

Welcoming homecare services into their home also challenged parents' identity. They reported that the boundaries between their private home life and a care facility were blurred, especially in shared places such as the living room. Thus, the limited size of their homes was challenging, with healthcare professionals using the living room as their workspace, invading parents' privacy:

If my husband and I are in the middle of something, like watching a movie in the evenings and the night shift worker arrives, we turn the tv off and go to bed. So, it is very good for our sleep rhythm, but it does mean that we give up our living room. (ID 1)

Several families renovated their homes or attempted to improve their spatial functionality. Although most parents attempted to create distinct zones within their homes to maintain their privacy as a family, the omnipresence of their child's needs was challenging:

He was constantly crying and throwing up his food, so we needed to keep an eye on him. Gradually, we were provided with night nurses at home. It helped somewhat, but since we lived in an 1870s house, you could hear the crying anyway. (ID 11)

Contrastingly, smaller houses proved to be advantageous for parents, as they would leave doors open or sleep in their children's room to monitor them closely.

Most parents sought to blend in, seeking places they could relax. Their well‐being often fluctuated in unfamiliar settings, particularly in public spaces where they felt observed by others. They yearned for recognition, solidarity, and a sense of belonging. Thus, many parents turned to social media platforms where they could freely express themselves and connect with others with similar experiences. This was exemplified by a father who described social media as a safety net (ID 14) and a mother who considered her chat group with other diabetes mothers to be invaluable for venting her feelings. However, some parents entered online spaces with caution due to their perceived potential risk of being exploited or bullied:

I do not think it is wise to go public as a caregiver on social media. some of the things that have sent me on an emotional roller coaster are other people who are thoughtless. They say and do things that sometimes are well‐intentioned and, other times, just offensive. (ID 2)

Being in nature offered a tranquil and existential space that allowed parents to distract themselves from their caregiving obligations. A father described how he was able to shift focus and create a feeling of *being in another world* amidst nature (ID 14). Nature alleviated stress and strengthened family bonds, allowing for joyful experiences for ill children, which brought a sense of relief and happiness to the parents. Nevertheless, some parents confided that they avoided outdoor places to prevent possible worsening of their children's illness.

### 5.3 Lived Time: “Time Does Not Seem to Exist”

The dynamic nature of time is perceived differently by different parents according to their experiences of events: their memories, the present, and their future. A variety of temporal aspects exist in these broad senses of time. Hence, the unpredictable and burdensome nature of caregiving challenges parents' perception of continuity in time. Some parents expressed that “time does not seem to exist” (ID 4) or that “time, in a way, passes both very quickly and very slowly at the same time; time is somehow just one big mush” (ID14). These expressions may reflect their experiences of an ambiguous time. Moments appear to stretch on endlessly in caregiving tasks yet, looking back, time seems to have flown by quickly. Such temporal dissonance is a lived reality that deeply influences parents' well being.

Parents with children suffering from severe conditions often expressed despair due to the intense caregiving responsibilities and a persistent shortage of time and energy. This was particularly evident in their interactions with healthcare professionals, another time‐consuming aspect of caring for their children. Parents tried to cope by developing routines and sharing caregiving duties. However, the necessity to prioritise the well being of their children left little time for anything else:

Daily life is the easiest. During the weekends, when most people rest, I have extra‐long night shifts, as is the case during Easter holidays, Christmas holidays, and other festive seasons. In those times, we have extra work, we eat sweets, and break our routines. Then, it becomes even more challenging. So, when others recover, I struggle. I never have time to relax. It is 24/7. (ID 1)

Routines and time management were also important when coordinating long trips between specialised healthcare services and home. Preparing for travel necessitated a balance between parents' subjective perception of time against the rigid time of appointments scheduled. Despite their best efforts, delays were often inevitable due to external factors such as weather conditions, caring arrangements for other siblings, and consideration of whether the sick children's health could endure a physically demanding journey:

The distance is already long, but it becomes even longer when you have a chronically ill child who does not sleep in her car seat and sits uncomfortably. When we last drove there, she had breathing problems halfway through, which made the trip extra long. (ID 1)

To avoid stressful and time‐consuming travels, parents preferred online meetings whenever possible. The lack of local healthcare services, particularly in rural areas, left parents feeling abandoned when handling complex care issues. After devoting considerable time to demanding technical procedures, one mother recounted her uncertainty about inserting a feeding tube without sufficient training.

Parents invested significant time and effort to normalise family life. Some described a proactive approach, preparing for potential shifts in the situation. Prioritising their time strategically helped. However, others coped by focusing on the present and taking each day as it came. Eventually, most parents adjusted to the ongoing changes, establishing a sense of stability within their family dynamics. Nonetheless, as parents aged and experienced declining health, they contemplated their future capacity to provide care:

I wonder if I ever will let her go completely? When I retire and she is an adult woman, will I still monitor her app? And then call her or send her text messages when her blood sugar levels are too high? How long will I keep doing that? (ID 6)

Although parents had to put their own plans on hold, some also expressed hope for the future, speaking of the possibility of medical advancements, adequate care provision, and increased independence for their children. Future expectations served as a source of motivation, providing courage despite their current challenges:

We still have hope that she will get better […] I'm looking forward to when she can sit on her own […] Those are things I'm looking forward to so that life will be less exhausting, not only regarding the progress itself. (ID 1)

Parents also described how spending time with their ill children had changed their values and broadened their life perspective. Through their children's illness trajectories, they uncovered previously unknown strengths, deeper empathy for others, and an appreciation of what really matters in life.

### 5.4 Lived Self‐Other: Parents' Changing Relationships

Parents described various social interactions ranging from intimate bonds with family and friends to more distant encounters with healthcare providers. The interviews revealed the profound impact of existential relationality on parents' relationships with others and on their overall well‐being. Their ability to maintain meaningful relationships was crucial in not only building existential strength but also shielding themselves from loneliness and worries.

Parents' marital relationships were adversely affected by their children's illness. They recognised the need to adjust their roles and emphasised empathy and generosity towards their spouses, stating: “We are in this together” (ID 1) and “I know you are doing your best” (ID 13). They seldom focused on their marital relationship; instead, they functioned as a cooperative team:

It is just a matter of managing the practicalities. The only discussion we have is about blood sugar levels and school and how we will handle it. There is no time for anything else. (ID 6)

Deprioritizing marital relationships could lead to feelings of resentment and tension. One parent stated, “the mortgage holds the relationship together more than love” (ID 14). Despite this, all parents acknowledged their mutual dependence and emphasised the necessity of taking turns and sharing responsibilities. Frustration and despair emerged when communication failed, especially if one spouse remained silent about their sufferings while the other tried to communicate. Some couples sought professional assistance to solve problems and strengthen their marital bond.

However, many parents realised the need to devote more attention to their marriages and prioritised spending time with their spouses. Unfortunately, the demanding nature of caregiving, for some parents, led to insurmountable strains on the marriage, resulting in separation or divorce:

We drifted apart; I think that the child's illness is the reason for that. We tried to save our marriage, as we believed that our daughter should not have divorced parents and cancer. (ID 4)

Parents worked hard to build a trusting relationship with their ill children. A mother recounted tearfully how she struggled to do so:

Despite my love for her and desire to take care of her, it felt more like a mission. For a long time, it seemed like it did not matter who comforted her. Then, she might as well be cared for by a healthcare worker. (ID 7)

To compensate for the children's suffering, some parents demonstrated an excessive level of care and protection. Parents continually watched over their children, striving to empower them to be courageous and stand up for themselves …

Several parents noted that their bond with their ill and healthy children differed, with some even observing significant changes in their relationship with their healthy children. Not all parents realised that their healthy children shielded their feelings by pretending everything was fine.

She perceived that we loved her less. I was shocked when she said her brother received a lot of attention and that we spent all our time with him. I had to sit down and reflect, and I could understand her. (ID 12)

Despite this, healthy children were often considered as reliable companions and sources of security due to their extensive knowledge of their ill siblings' condition. However, parents were cautious about overburdening them with caregiving responsibilities. Therefore, parents frequently turned to their extended family, especially the grandparents, for emotional and practical support, including household chores, transportation, and assistance with school‐related issues. Grandparents' assistance was an absolute necessity and a priceless contribution to family functioning.

Their children's illness affected the parents' ability to maintain close friendships. As one parent stated: “We have lived completely isolated for 10 years. We have hardly any friends left” (ID 12). Some parents kept their struggles private, believing that friends lacked understanding of how their children's illness impacted their daily life. They also found their friends' issues trivial in comparison. However, they concealed their challenges to appear normal and avoid seeming like they were complaining. Thus, few parents got support from friends. Although they missed these social bonds, they highlighted the benefits of connecting with parents in similar situations, which alleviated their loneliness.

One becomes both tired and exasperated, and then it is helpful to talk to someone who understands you. Someone who is experiencing the same problems. (ID 5)

Parents described their relationship with healthcare professionals as essential for their children's well‐being. They especially remembered those who treated their children with dignity and respect and provided personalised and compassionate care.

We encourage them [healthcare personnel] to follow their natural instincts. Hold her and cuddle her when she needs it. We feel comforted and secure knowing that she is in good hands. (ID 7)

Parents expressed a desire to develop trusting mutual relationships with healthcare professionals, emphasising the importance of shared decision‐making and being well‐informed about their children's condition and treatment plans. However, they raised their voices when crucial information was not conveyed, or proper care was not provided:

So, when I say that it should be done this way and that I have done this before, I have been doing this for a year. I knew exactly how it should be performed. I expected them to listen to me. (ID 4)

Although parents made efforts to ensure healthcare professionals felt comfortable and valued, understanding the crucial role they played in alleviating their burdens, they occasionally felt that the professionals were less attentive to their existential needs. They longed for opportunities to have more meaningful conversations about their experiences and concerns. Some parents mentioned that conversations with their general practitioner provided comfort and courage:

I was so worn out that I would find myself on the verge of tears just standing outside a grocery store, unable to focus on what food to purchase. When I mentioned this to my doctor, he said, “You know that is not normal.” I explained that my daughter barely sleeps and that I am overwhelmed with work, yet I insisted that things would get better. My doctor replied, “Life shouldn't be this way. You should not be crying before you enter a grocery store”. (ID 9)

## 6 Discussion

This study sheds light on how existential challenges are intensified when parents face extreme caregiving demands. Our findings reveal that confronting these existential struggles could lead to new meaning in life. Meaning arises from believing in something and feeling whole and connected to life (Arredondo and Caparrós 2023). We observed that all parents in our study reported substantial shifts in their sense of self. Bally et al. (2018) have similarly noted that parenting a child with a severe illness is a crisis, involving a continual search for purpose in adjusting and coping. Our findings emphasise that the emergence of hope is crucial for experiencing meaning and establishing a new normality. Binder (2022) further explains that existential well‐being can act as a buffer against illness and enhance health. Our findings suggest that nurses are positioned to recognise the complexities of parents' existential experiences, and if parents are met with compassionate care, existential suffering can ignite personal strength and foster wellbeing, consistent with the meta‐synthesis by Nygård and Clancy (2018).

Grounded in van Manen's (2023) framework, our study provides novel insights into how parents' existential experiences are influenced by the dimensions of corporality, spatiality, relationality, and temporality, within their lifeworlds, contributing to the knowledge of families caring for a child with CCNs. Recognising the distinct characteristics of each existential dimension, we examined them separately, acknowledging that changes in one can affect others and, consequently, parents' lived experiences. The four identified themes underscore the complexity of each existential dimension, enhancing our understanding of the existential needs of parents raising children with CCNs.

Parents relentlessly strive to preserve their family's existential well being while managing the fluctuating, intense caregiving demands, often hindered by the scarcity of necessary resources. They assume the roles of care experts and advocate for their children's well being. Yet, they frequently encounter gaps in their knowledge and skills to effectively navigate the existential challenges in parenting roles. Our findings reveal that caring for ill children is emotionally demanding and parents struggle to let go of constant worries, intensifying their sense of an unbearable and at times forced existence. This strain manifests as significant parental stress and exhaustion, aligning with international findings from Yang et al.'s (2024) umbrella review, which contends that such caregiver stress directly impairs parents' capacity to care for their children, subsequently influencing the children's treatment and rehabilitation trajectory.

Parents' descriptions of emerging physical symptoms resonate with Coughlin and Sethares's (2017) descriptions of chronic sorrow, described as cyclical, progressive, recurrent, and persistent feelings, often intensifying as children grow or their illnesses relapse. This emotional burden, coupled with existential feelings of loss and loneliness, challenges the parents' sense of freedom and belonging. Our research illuminates not only the physical and emotional strain experienced by parents but also the burden of coping with the unpredictable nature of their children's illnesses. Uncertainties amplify worries about their children's health and its impact on family dynamics, financial stability, and personal well being. This aligns with Currie and Szabo's (2019) research that unpredictability can cause sensations of bodily ambiguity and disrupt parents' sense of control.

van Manen (2023) characterises parental worries as a specific form of caring—an existentially rooted responsibility to their child—aligning with our findings that children's illnesses may impact parents' existence. van Manen (2023) refers to this as “care‐as‐worries,” particularly heightened during children's difficulties or illness. We found that parents exhibit an intense level of care and vigilance, a dedication that stands out from their other relationships, demonstrating an exceptional sense of commitment toward their children. Such profound caregiving takes place within environments and frameworks that support genuine caring relationships (van Manen 2023), indicating that nurses ought to be fully present and attentive, actively listening, and showing genuine interest in the thoughts and feelings of others (Pratt, Moroney, and Middleton 2021). This reflects our findings indicating that parents are more content in their caregiving role when they are supported by well‐coordinated services, appropriate information, and emotional and practical support. Other similar studies have identified that these needs may vary across diagnoses and treatment stages (Thomas et al. 2023).

The capacity to care for others is compromised when one's personal life is impacted, emphasising the critical need to prioritise self‐care. Nevertheless, parents place immense expectations on themselves, exacerbated by feelings of excessive caregiver guilt. This is consistent with the findings of Masefield et al. (2020), who demonstrated that such feelings could lead to anxiety and depression. Despite these challenges, our findings indicate that parents also recognise the positive aspects of caregiving, fostering resilience, and pursuing personal growth, as echoed in the study of Palacio et al. (2020).

Despite parents' efforts to encourage themselves to do their best, their bodies often betray them by showing signs of exhaustion and the urgent need for respite care and personal time. Respite care can effectively enhance parents' mental health and sustain their caregiving capacity (Azimi et al. 2024). In our findings, most parents initially felt ambivalent about leaving their ill children and were reluctant to use respite care services. However, in the long run, they recognised the benefits and welcomed a temporary relief from caregiving, placing their trust in the healthcare professionals providing respite care. This contrasts with prior research (Murphy et al. 2022), which indicated that respite services are underused, lack competent personnel, and fail to meet the comprehensive needs of families.

The novelty of our findings is that they demonstrate that parents' existential needs are interwoven throughout their lifeworlds, impacting various aspects of the existential dimensions of corporality, temporality, spatiality, and relationality. Nurses' awareness of the broad spectrum of the unique needs of parents, including how they are expressed, is essential to deliver high quality care (Nygård and Clancy 2018). Specifically, our findings underscore the need for available, sensitive, and personalised care to foster caregiving resilience, in accordance with the findings of the study by Hynnekleiv et al. (2023). Drawing on the work of van Manen (1998), this reminds us of the fundamental ethos of nursing, which entails trying to understand the experience of others and suspending our preconceptions in favour of meeting the parent's existential needs. Sometimes, a reassuring conversation might create an open atmosphere for parents to vent their specific needs (van Manen 1998). Listening to their requirements may help reestablish their sense of attunement to their body and reconnect with the world around them (van Manen 1998), thereby emboldening them to carry on despite adversity.

### 6.1 Strengths and Limitations

This study presents rich descriptions of the existential lived experiences of 16 parents of children and young people with CCNs. During the interviews, parents shared their various experiences of parenting children and young people of various ages and with different diagnoses, symptoms, and needs. The parents involved in this study are recipients of health and social services across different regions of Norway, ensuring a geographically diverse and comprehensive sample. It is noteworthy that the complexity of children's and young people's needs is not static but dynamic and situational, changing over the course of their lives. The severity and impact of these needs are subject to fluctuations and influenced by each family situation and the healthcare system they navigate. Thus, the experiences of a parent of a child or young person with CCNs cannot be generalised to all other parents of children and young people with CCNs. However, the use of snowball sampling may have contributed to an unbalanced sample by overrepresenting certain characteristics. The homogeneity of the sample may present a limitation as the sample mainly comprises native Norwegians and only includes two fathers.

Nevertheless, this study's strength lies in the evocative nature of its findings, with parents' lived experiences being vividly quoted and situated within their specific contexts, enhancing the closeness to the phenomena explored. Thus, the provocative quality of phenomenological findings may trigger epiphanies, enabling readers to resonate with this phenomenon from a new and deeper perspective (van Manen 2023). By applying van Manen's (2023) lifeworld existential dimensions, valuable aspects of parents' lifeworlds were uncovered. Despite extensive searches, the authors have found no study that describes in depth how the various existentials interact and influence parents' existential lives. Additionally, this approach facilitated the structuring and analysis of parents' lived experiences, strengthening methodological congruence and consistency.

### 6.2 Recommendations for Future Research

While interviews are a commonly used method to explore parents' experiences of their lifeworlds, they may not capture the full depth and complexity of these experiences. There is a dearth of published research that adopts an existential and phenomenological perspective using observational methods. Globally, most parents provide care for children with CCNs at home, making lived observation designs (van Manen 2023) crucial for future research. This approach will enable a deeper exploration of how life unfolds in family homes. According to our findings, the parents' home not only facilitates family bonds and autonomy but also entails restrictions, insecurity, and confinement. Employing an observational research design may yield profound insights and capture nuanced lived descriptions of the interactions not only among family members but also between nurses, ill children, their healthy siblings, and their parents within the homecare setting, revealing insights that interviews may not uncover.

## 7 Conclusions and Implications

This study offers novel in‐depth insights into the existential experiences of parenting children with CCNs and the impact on parents' well being, emphasising its relevance for clinical nursing. Parents employ various strategies to build the courage and persistence needed to confront existential adversities, underscoring the need for nurses to engage with and respond to parents' lived descriptions of their lifeworlds. Yet, our findings clearly reveal that healthcare professionals do not always address these existential experiences, which are crucial for parents to sustain their caregiving demands.

Employing van Manen's phenomenology of practice approach contributes to a more original and comprehensive understanding of these parent's lives, which could enrich nurses' perceptiveness of parents' existential experiences, fostering a more reflective nursing practice. Nurses, present during intimate life moments, should engage in meaningful dialogue with parents, recognising that the insights gained from these interactions aimed at helping parents achieve existential growth. This phenomenological perspective calls for a genuine nursing presence characterised by an attitude of open sensitivity and attentive listening to parents' subjective experiences. Our findings suggest that nurses working with such families should be aware of parents' expressions of shifting relationships, feelings of entrapment, and temporal uncertainties. Most importantly, nurses should be especially attuned to parents who report feelings of inadequacy, caregiver guilt, anxiety, and loneliness, as these emotions can be life‐constraining. Thus, our findings can improve nursing education and practice by preparing nurses to respond competently to parents' needs. Internationally, with paediatric care moving from hospitals to homes, healthcare policies should create framework conditions for nurses to address existential issues effectively.

## Author Contributions

In accordance with the guidelines of the International Committee of Medical Journal Editors, all authors significantly contributed to the study's conceptualization, design, data acquisition, analysis, and interpretation. C.N. authored the initial draft of the manuscript, which was critically revised for intellectual content by A.C. and G.K. All authors participated sufficiently in this research to take public responsibility for the content and agreed to be accountable for all aspects of the work, ensuring that questions related to the accuracy and integrity of any part of the manuscript are appropriately investigated and resolved. All authors have approved the final version of the manuscript for submission.

## Conflicts of Interest

The authors declare no conflicts of interest.

### Peer Review

The peer review history for this article is available at https://www.webofscience.com/api/gateway/wos/peer‐review/10.1111/jan.16509.

## Supporting information


Appendix S1.

## Data Availability

The datasets generated during and/or analysed during the current study are not publicly available due to ethical considerations but are available from the corresponding author upon reasonable request.
